# The Mutation in *wbaP cps* Gene Cluster Selected by Phage-Borne Depolymerase Abolishes Capsule Production and Diminishes the Virulence of *Klebsiella pneumoniae*

**DOI:** 10.3390/ijms222111562

**Published:** 2021-10-26

**Authors:** Marta Kaszowska, Grazyna Majkowska-Skrobek, Pawel Markwitz, Cédric Lood, Wojciech Jachymek, Anna Maciejewska, Jolanta Lukasiewicz, Zuzanna Drulis-Kawa

**Affiliations:** 1 Laboratory of Microbial Immunochemistry and Vaccines, Ludwik Hirszfeld Institute of Immunology and Experimental Therapy, Polish Academy of Sciences, 53-114 Wroclaw, Poland; marta.kaszowska@hirszfeld.pl (M.K.); wojciech.jachymek@hirszfeld.pl (W.J.); anna.maciejewska@hirszfeld.pl (A.M.); 2 Department of Pathogen Biology and Immunology, University of Wroclaw, 51-148 Wroclaw, Poland; grazyna.majkowska-skrobek@uwr.edu.pl (G.M.-S.); pawel.markwitz@uwr.edu.pl (P.M.); 3 Department of Microbial and Molecular Systems, KU Leuven, 3001 Leuven, Belgium; cedric.lood@kuleuven.be; 4 Department of Biosystems, KU Leuven, 3001 Leuven, Belgium

**Keywords:** *Klebsiella* phage, capsule degrading depolymerase, phage resistant mutant, capsular polysaccharide

## Abstract

*Klebsiella pneumoniae* is considered one of the most critical multidrug-resistant pathogens and urgently requires new therapeutic strategies. Capsular polysaccharides (CPS), lipopolysaccharides (LPS), and exopolysaccharides (EPS) are the major virulence factors protecting *K. pneumoniae* against the immune response and thus may be targeted by phage-based therapeutics such as polysaccharides-degrading enzymes. Since the emergence of resistance to antibacterials is generally considered undesirable, in this study, the genetic and phenotypic characteristics of resistance to the phage-borne CPS-degrading depolymerase and its effect on *K. pneumoniae* virulence were investigated. The K63 serotype targeting depolymerase (KP36gp50) derived from *Klebsiella* siphovirus KP36 was used as the selective agent during the treatment of *K. pneumoniae* 486 biofilm. Genome-driven examination combined with the surface polysaccharide structural analysis of resistant mutant showed the point mutation and frameshift in the *wbaP* gene located within the *cps* gene cluster, resulting in the loss of the capsule. The sharp decline in the yield of CPS was accompanied by the production of a larger amount of smooth LPS. The modification of the surface polysaccharide layers did not affect bacterial fitness nor the insensitivity to serum complement; however, it made bacteria more prone to phagocytosis combined with the higher adherence and internalization to human lung epithelial cells. In that context, it was showed that the emerging resistance to the antivirulence agent (phage-borne capsule depolymerase) results in beneficial consequences, i.e., the sensitization to the innate immune response.

## 1. Introduction

*Klebsiella pneumoniae* belongs to the bacterial ESKAPE group (*Enterococcus faecium*, *Staphylococcus aureus*, *Klebsiella pneumoniae*, *Acinetobacter baumannii*, *Pseudomonas aeruginosa*, and *Enterobacter* spp.) and is placed on the top priority WHO list of “critical” pathogens and a target for the development of therapeutic strategies combating hypervirulence and multidrug-resistance problems [1,2,3]. The cell surface-associated polysaccharides and glycolipids, namely capsular polysaccharides (CPS also known as K-antigen) and lipopolysaccharide (LPS; O-antigen), as well as the exopolysaccharides (EPS) are the major virulence factors of *K. pneumoniae* [4,5]. *Klebsiella* strains can effectively be discriminated against based on the O- and K-typing. To date, 11 O-serotypes have been identified for *K. pneumoniae* (O1, O2a, O2ac, O2afg, O2aeh (called also O9), O3, O4, O5, O7, O8, and O12), with two sub-serotypes (O3a, O3b) [6]. O1 and O2 O-PS structures are composed of galactan-I (O1 and O2) and galactan-II (O1) that are characterized by at least two structural variants (v1 or v2), i.e., O1v1 or O1v2 and O2v1 or O2v2 [6]. In turn, there are more than 140 genetically distinct capsular locus types, of which 77 well-characterized chemical structures are used in serotyping [7]. The O-antigen is synthesized stepwise in the cytosol, further assembled, and exported through sophisticated transportation machinery onto the bacterial surface. Among three known O-antigen synthesis pathways—(i) the ATP-binding cassette (ABC) transporter-dependent, (ii) the Wzx/Wzy-dependent, and (iii) the synthase-dependent system [8,9]—*K. pneumoniae* utilizes the first system. In turn, the CPS is produced via a Wzx/Wzy-dependent pathway [10], identical to *Escherichia coli* group I CPS synthesis [10,11,12,13]. Although a Wzx/Wzy-dependent pathway could be also utilized for EPS surface expression in *E. coli* [14], there is no information available regarding EPS-secretion strategy in *K. pneumoniae*. Despite the differences in modes of the O-antigen and CPS export, their initial step in the biosynthesis proceeds according to the similar scheme, using the sugar specific glycosyltransferases [4,15,16,17]. This process involves the formation of sugar-pyrophosphate undecaprenol (sugar-Und-PP) by transfer of a sugar monophosphate (sugar-1-P) to the universal lipid-carrier moiety (undecaprenyl phosphate, Und-P), at the cytoplasmic face of the inner membrane, thereby initiating polymers formation. In essence, the *Klebsiella* O-antigen repeating units biosynthesis is initiated by the transfer of N-acetylglucosamine (GlcNAc)-1-P onto Und-P via GlcNAc-1-phosphate transferase (WecA), yielding undecaprenyl-diphosphate-N-acetyl-glucosamine. In turn, the CPS synthesis initiation, depending on its type, is catalyzed by the initial glycosyltransferase, WbaP or WcaJ that transfers galactose-1-phosphate or glucose-1-phosphate, respectively, onto Und-P and is further processed by serotype-specific transferases [7,12,13]. The capsule is ultimately transported and covalently or non-covalently linked to the outer face of the outer membrane, whereas EPS is loosely attached to the surface, and LPS is linked by lipid A.

The virulence factors, both the CPS and O-antigen allow *K. pneumoniae* to avoid host defenses, suppress the early inflammatory response, and enhance adherence and biofilm formation [18,19,20,21]. They also protect bacteria from abiotic stresses, including UV light, desiccate as well as lower the sensitivity to antibiotics and antimicrobial peptides, and hinder phages access, thereby preventing the viral infection [22,23,24]. On the other hand, polysaccharides may constitute a target for specific phages that are evolutionarily adapted to overcome the glycan structures by virion-associated enzymes (lysins and depolymerases) in order to infect bacterial cells [25]. The latter digest with high specificity the CPS/LPS/EPS layers sensitizing bacteria to environmental stress and the host immune system. It has been shown that capsule-degrading depolymerases effectively reduce the virulence, making bacteria more prone for eradication by immune response mechanisms [26,27,28]. Disarming targeted pathogens rather than eradicating them by broad-spectrum antibiotics is considered an effective and evolutionarily robust way to manage infections [29,30]. Importantly, this approach provides antimicrobials that simultaneously overcome two serious adverse effects: drug resistance emergence and commensal dysbiosis. Currently, little is known about the resistance to antivirulence therapy based on depolymerases; thus, it requires comprehensive studies for its elucidation and assessment.

As the risk of resistance development is perceived as an adverse component of any treatment, we decided to examine the phage depolymerase-driven resistance in the model of *K. pneumoniae* serotype K63. Our recent studies showed that the resistance to capsule-targeting *Klebsiella* phages is based on receptor modification and involves both mutations in the capsule biosynthesis cluster (*cps*) or qualitative changes in EPS [31]. In this study, the K63 capsule degrading recombinant depolymerase (KP36gp50) derived from *Klebsiella* siphovirus KP36 was used, which was previously identified and characterized as a promising antivirulence agent [27] to select resistant mutants from the *K. pneumoniae* serotype K63 population. Additionally, the genetic background and biochemical processes relevant to *Klebsiella* pathogenicity were investigated in depolymerase-resistant mutant.

## 2. Results

### 2.1. Phenotypic Characterization of Kp486 Mutant Resistant to Recombinant Depolymerase KP36gp50

**Figure 1:**
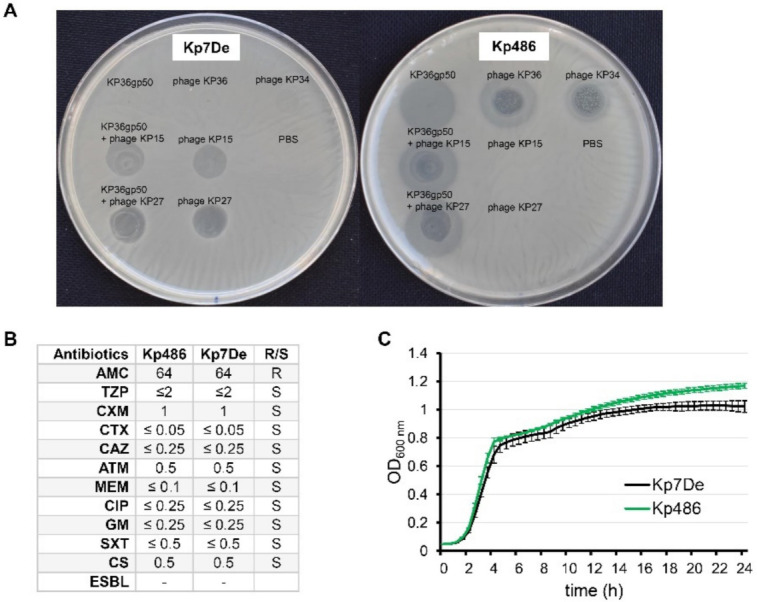
Phenotypic characterization of Kp486 mutants resistant to recombinant depolymerase KP36gp50 versus the wild-type strain. (**A**) The spot test with KP36gp50 depolymerase (2.5 µg) and phages (KP36, KP34, KP27, and KP15) (5 × 10^5^ PFU) on *K. pneumoniae* Kp7De mutant and its parental Kp486 strain. Lack of the translucent halo zones and clear plaques on the Kp7De lawn following spotting of depolymerase, and phages KP36 and KP34 respectively, indicate that Kp7De lost susceptibility to K63 capsule-targeting agents; (**B**) Antibiotic susceptibility profiles of Kp7De and Kp487 expressed as MIC (mg/L) (R-resistant and S-susceptible); (**C**) The fitness characteristics as proxied by the growth rate of Kp7De and Kp486 strains are shown in growth curves with the mean ± SEM of three independent experiments.

While studying the antibiofilm potential of recombinant K63 capsule-degrading depolymerase (KP36gp50), phenotypically smaller *K. pneumoniae* Kp486 colonies were isolated, which turned out to be both resistant to KP36gp50 activity as well as to phages targeting K63 receptor (siphovirus KP36 and podovirus KP34) (Figure 1A). For further genotypical and virulence analyses, one isolate (hereafter as Kp7De) was selected. Interestingly, Kp7De had gained sensitivity to myoviruses KP15 and KP27 that recognize an alternative receptor, likely of proteinaceous nature, which is similar to previously described KP36-induced mutants [31]. Phages KP15 and KP27 also formed plaques on Kp486 and Kp7De lawns previously spotted with KP36gp50. As the cell-surface alterations can affect the susceptibility of bacteria toward antimicrobials, the antibiotics susceptibilities of both isolates were assessed (Figure 1B). The antibiotic susceptibility profile of the mutant has not been changed compared to the wild-type strain. Furthermore, the growth kinetics revealed the superimposed curves of Kp486 and Kp7De, suggesting that the resistance to the depolymerase activity and K63 capsule-targeting phages was not associated with higher fitness costs (Figure 1C).

### 2.2. Mutation in the cps Locus Confers Depolymerase Resistance

**Figure 2:**
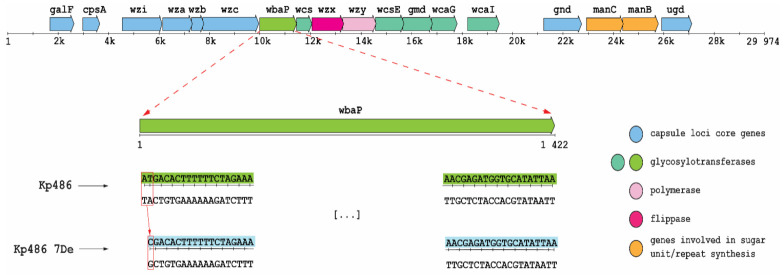
Genetic organization of K63 *cps* region in Kp486 strain and a frameshift mutation in Kp7De mutant. Arrows indicate the direction and relative length of open reading frames (ORFs). Base deletion and substitution (box) were observed in the start codon of *wbaP* gene in Kp7De.

To identify the genetic basis of resistance selected by KP36gp50, whole-genome SNP and mapping analyses of Kp7De were performed against the parental Kp486 strain genome. As shown in Figure 2, Kp7De mutant harbored a mutation in the *wbaP* gene located in the *cps* locus i.e., a combination of a deletion with a substitution causing a replacement of two nucleotides (adenine and thymine) with a single cytosine. This causes a change of reading frame in the start codon of the gene encoding WbaP glycosyltransferase responsible for the transfer of galactose-1-phosphate to the undecaprenyl phosphate acceptor moiety (Und-P). As such, the initiation of the synthesis of the CPS repeating unit was eliminated [7,12,13]. In addition, three synonymous mutations and two mutations in the intergenic region were detected in the Kp7De isolate (Table S1). One of those mutations is positioned in the possible promoter of the putative L-galactonate transporter.

### 2.3. Lack of Functional WbaP Abolishes Capsule Production

To confirm the consequences of genetic modification in the *cps* cluster, *K. pneumoniae* strains Kp486 and Kp7De were examined for the presence of main surface antigens such as LPS (O-antigen), CPS (K-antigen), and EPS, and further compared to genotypes of O and K antigens analyzed using Kaptive Webtool (Table 1). For phenotype analyses, LPS and CPS/EPS were isolated from Kp486 and Kp7De strains by the hot phenol/water extraction (LPS) or according to Bales’s protocol (EPS/CPS). Hydrolyzed LPS and crude polysaccharides (CPS/EPS) were fractionated by size exclusion chromatography and analyzed by NMR spectroscopy. The yields for LPS isolation from dried bacterial cells were 0.7% for Kp486 and 5.3% for Kp7De, with a 7.6-fold higher value for a mutant isolate. Contrary to the wild-type strain Kp486, no CPS was identified in Kp7De (Table 1). Moreover, no EPS was identified for both strains in preparations obtained according to Bale’s protocol.

**Figure 3:**
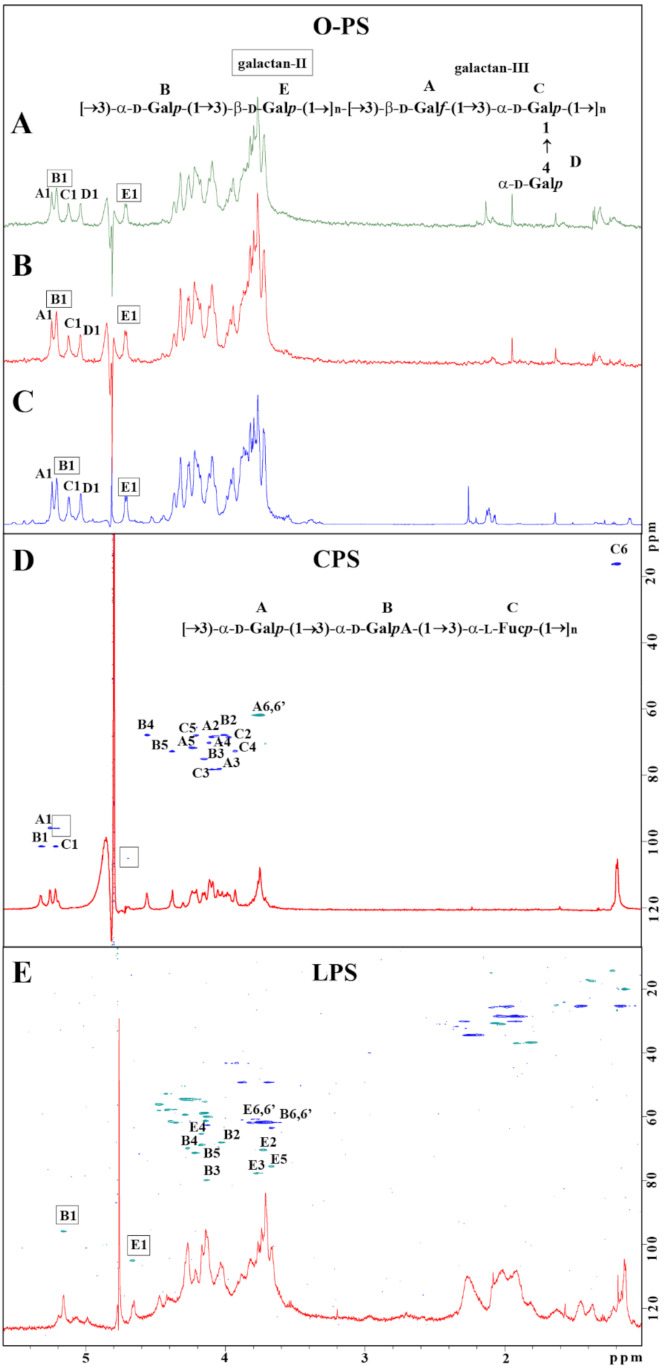
Structural analysis of carbohydrate surface antigens of *K. pneumoniae* Kp486 and Kp7De. ^1^H NMR spectra of O-antigens isolated from Kp486 (**A**) and Kp7De (**B**) compared with the reference spectrum of O1v2 antigen (strain Kp24) (**C**). ^1^H, ^13^C HSQC-DEPT NMR spectra overlaid with ^1^H NMR profiles of the polysaccharides isolated from strains Kp486 and Kp7De identified as CPS (**D**) and LPS O1v2 (**E**), respectively. NMR spectra were acquired at 298 K. Repeating units of O1v2 (**A**) and CPS K63 (**D**) antigen were shown as inset structures. The capital letters refer to carbohydrate residues as described in the inset structures and Table 2 (K63) and Table S2 (O1v2 O-PS).

For LPS O-serotyping, Kp486 and Kp7De LPS (5 mg) were degraded by mild acid hydrolysis, and O-PS was isolated by gel permeation chromatography on TSKgel G2500PW column. Three fractions were eluted (1–3) from both LPS and checked by ^1^H NMR spectroscopy. Fractions 1 did not contain sugar constituents, fractions 2 were identified as O-PS, and fractions 3 were identified as core oligosaccharides of LPS. Comparison of the ratio between peak areas of fractions 2 and 3 revealed a higher quantity of the O-PS in Kp7De, indicating the presence of longer O-PS in the mutant strain (Figure S1). ^1^H and ^1^H,^13^C HSQC-DEPT NMR spectra were used for O-serotype identification by preliminary comparison with the library of NMR spectra for O1 (variant 1 and 2), O2a, O2afg, O2aeh, O3, O3a, O3b, O4, O5, O7, O8, and O12 O-antigens followed by structural analysis. O-PS isolated from strains Kp486, and Kp7De revealed O1v2 serotype (Figure 3A,B) identical with O-PS isolated from reference *K. pneumoniae* strain Kp24 [33] (Figure 3C), which was demonstrated by comparison of ^1^H NMR spectra (Figure 3A–C) and verified by complete spectra interpretation for Kp486 (Table S2). All O-antigen phenotypes confirmed the presence of smooth-type LPS of O1v2 O-antigen structure that was in agreement with the genotype analyzed by Kaptive Web (Table 1).

For K-serotyping, polysaccharides isolated from Kp486 and Kp7De according to Bales’ protocol were fractionated on TSKgel G3000PW column. Fractions were checked by ^1^H and ^1^H,^13^C HSQC-DEPT NMR spectroscopy showing the presence of a major polysaccharide fraction. Interpretation of NMR spectra for Kp486 showed three sugar residues (A, B, C) attributed to the K63 repeating unit structure (Figure 3D, inset structure) that was in agreement with previously published data [34,35,36] as well as additional low-intensity signals indicating the presence of another polysaccharide (Figure 3D, signals marked by squares). The repeating unit structure of K63 was identified as →3)-α-D-Gal*p*-(1→3)-α-D-Gal*p*A-(1→3)-α-L-Fuc*p*-(1→, where sugar constituents were assigned as A, B, and C, respectively (Figure 3D). Detailed analysis of K63 CPS is described in Table 2. There were differences compared to previously published data for the K63 capsule [34,35], CPS K63 isolated from strain Kp486 was devoid of formyl and O-acetyl groups. Contrary to strain Kp486, the preparation of Kp7De mutant revealed the presence of B and E sugar residues represented by two anomeric signals B1 and E1 and was devoid of K63 CPS (Figure 3E). Detailed analysis of NMR spectra collected for Kp7De polysaccharides indicated the presence of signals characteristic for LPS containing O1v2 O-specific polysaccharide only, with predominating signals of the galactan-II (E and B residues) (Figure 3E and the inset structure in Figure 3A). Moreover, NMR spectra showed the presence of fatty acid residues that indicated separate lipid contamination or the presence of complete O1v2 LPS molecules (including lipid A moiety).

### 2.4. Capsule Loss Due to wbaP Mutation Reducing the Virulence of K. pneumoniae

**Figure 4:**
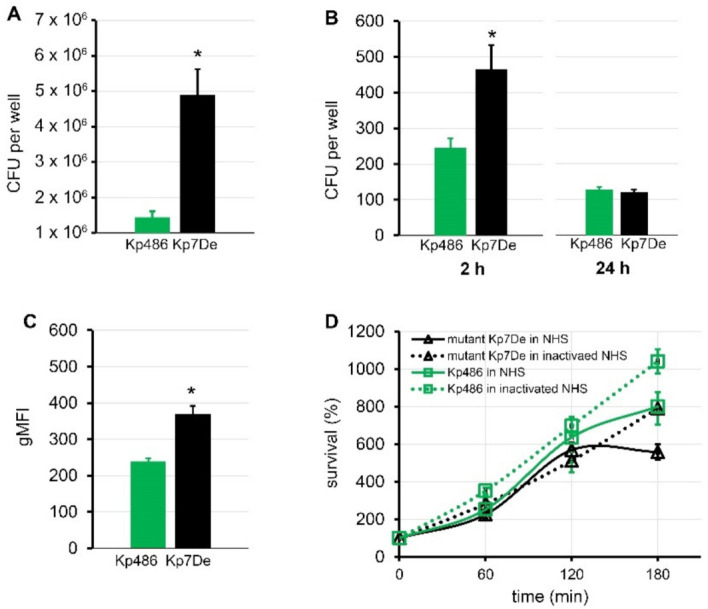
Comparison of virulence of capsule-deficient Kp7De mutant and its parental Kp486 strain. Adhesion (**A**) and time-dependent internalization (**B**) by the A549 lung epithelial cells expressed in CFU per well and shown as the mean ± SEM (*n* = 4). (**C**) Phagocytosis by the human monocyte cell line THP1 determined by flow cytometry. UV-killed FITC-labeled bacterial cells were mixed with monocytes at a 100:1 ratio. Quantification of cellular uptake of bacterial cells by phagocytes is expressed as the geometric mean fluorescence intensity (gMFI) from the gated sample with phagocytosing cells ± SEM (*n* = 3). (**D**) Time-dependent bactericidal effect of 50% NHS or heat-inactivated NHS for 3 h at 37 °C. The percentage of survival was determined as the number of bacteria that survived relatively to the initial bacterial loading (*n* = 3). Asterisks indicate a statistically significant difference (*p* < 0.05).

It is generally accepted that CPS protects bacteria from the innate host response, including phagocytosis and/or complement-mediated killing [5,20]. Moreover, encapsulated *K. pneumoniae* strains have been shown to associate more intensively with some epithelial cell lines and macrophages [37,38]. To address the role of the dysfunctional WbaP in the host–pathogen interaction, the ability of the Kp7De mutant in terms of adherence and internalization to human lung epithelial cells (A549) and phagocytes (THP-1), as well as its ability to survive serum exposure, were examined. As shown in Figure 4A, Kp7De showed significantly increased cell associations to the A549 cells compared to the wild-type strain (*p* < 0.0001), which confirms previous findings that CPS decreases bacterial adherence, likely through the masking of short proteinaceous adhesins [39]. To determine whether the loss CPS production would favor the invasion process, a gentamicin protection assay was performed using A549 cells. Two-fold higher intracellular bacterial reservoir for Kp7De after 2 h of incubation in a gentamicin-containing medium indicated that a capsule-deficient mutant invaded lung epithelial cells more efficiently compared to Kp486 (*p* < 0.0005, Figure 4B). With the prolonged incubation (24 h) of epithelial cells containing bacteria, the survival rates of intracellular Kp7De decreased nearly four-fold, while approximately half of the Kp486 cells were still viable. The results suggested that deficiency of *wbaP* in *K. pneumoniae* sensitizes bacteria to intracellular killing by epithelial cells. Furthermore, the phagocytosis assay performed with monocyte/macrophage cell line THP1 revealed that the capsule-defective mutant was more prone to phagocytosis with almost two-fold increased uptake compared to the parental strain (*p* ≤ 0.0005, Figure 4C). Conversely, lack of capsule in Kp7De did not correlate with the loss of serum resistance (Figure 4D). Both Kp7De and its parental strain survived the exposure to 50% normal human serum over a 3 h incubation period at 37 °C or even was able to propagate.

## 3. Discussion

The emergence of resistance to conventional drugs is an ever-present hurdle of each antimicrobial strategy [40]. However, the resistance to antivirulence agents, contrary to appearances, might be beneficial for therapy and leads in some cases to disarming the pathogen. In this study, it was aimed to address two major questions of how bacteria develop resistance to such agents and how those changes affect their virulence. The capsule-targeting depolymerase derived from *Klebsiella* phage was utilized as the selective stressor and investigated the resistance emergence implication for *K. pneumoniae* pathogenicity. Based on comprehensive genome-wide examination combined with the surface polysaccharides structural analysis, it was showed that point mutation (frameshift) in the first nucleotides of the *wbaP* gene located within the *cps* cluster in the Kp7De strain impaired capsule formation without reduction in relative fitness in terms of growth rate and generation time compared to its parental strain (Kp486). The in silico Kaptive tool indicated K63 and O1v2 serotypes in both strains, whereas the structural analysis revealed the O1v2 phenotype in both strains and the lack of K63 CPS in Kp7De. Contrary to previously published data [34,35], CPS K63 isolated from the Kp486 strain was devoid of formyl and O-acetyl groups. Since the Kp486 strain was sensitive to Kp36 phage and KP36gp50 depolymerase derived from *Klebsiella* siphovirus KP36, formyl and O-acetyl groups seem not relevant for the resistance mechanism to depolymerase. In addition, the sharp decline in the yield of CPS was accompanied by the production of a larger amount of LPS (7.6-fold higher yields of LPS extraction) with an identical composition to that in the wild-type strain. The possible contamination of Kp486 LPS preparation by CPS might result in a lower yield of O-PS isolation by mild acid hydrolysis. However, the elution profiles obtained for LPS-derived poly- and oligosaccharides fractionations indicated the higher ratio of unsubstituted core oligosaccharide to the O-PS fraction containing core oligosaccharide substituted with O-PS (Figure S1). Interestingly, in *E. coli*, the amount and ratio of different cell surface polymers, including the enterobacterial common antigen (ECA) and LPS O-antigen, are dependent on the Und-P cellular pool acceptor. As such, it is tempting to speculate that the Und-P not serving for the CPS synthesis was in turn used to form the O-antigen, thereby enhancing the production of LPS in Kp7De mutant forming longer and more abundant O-chains [41].

Since both CPS and LPS O-antigen are crucial determinants of medically relevant phenotypes, it was checked whether (and how) the alterations in those structures affect the pathogen–host interplay, including potential evasion of host immune response. Given the critical role of CPS in phagocytosis preventing [42,43], the susceptibility of Kp7De mutant to non-opsonic (lectino-)phagocytosis was assessed. As anticipated, the depolymerase resistance evolved by a mutation in the *cps* locus sensitized *Klebsiella* cells to phagocytosis by THP1 monocytes/macrophages. This observation is in line with our previous report showing most likely that the removal of the CPS barrier promotes the phagocytosis process, probably by an appropriate presentation and accessibility of ligands to phagocytic cell receptor [28]. Taken together, these data suggest that during *Klebsiella* infections, depolymerase application could selectively modify the bacterial population in favor of *cps* mutants prone to effective elimination by the innate host response. In this regard, depolymerases cause the resistance emergence, simultaneously diminishing the pathogenicity and increasing the immunogenicity of *K. pneumoniae*.

Another first-line defense mechanism of innate immunity is related to the complement-mediated lysis. Our study revealed that the loss of a CPS layer did not change the serum insensitivity of bacteria; thus, the next structure credited with the protective role against serum is LPS with specific O-antigen composition and the side chain length [44,45,46]. *Klebsiella* can also utilize LPS-containing outer membrane vesicles (OMVs) to saturate soluble complement proteins, resulting in the inhibition of complement cascade activation [47,48]. Finally, other complement evasion strategies, such as targeting host regulators of complement (e.g., factor H) cannot be excluded [49].

Since *K. pneumoniae* is a pathogen often causing pneumonia, the effect of depolymerase-driven *wbaP* mutation on lung epithelial cells colonization was also investigated. It turned out that mutant Kp7De adhered and invaded into the A549 cells more efficiently than its encapsulated parental strain. This could be due to either the higher availability of adhesins at the cell surface, previously hidden under the CPS layer, or their overexpression when capsule synthesis is turned off [38]. Consistent with those results, de Astorza et al. [50] proved that the internalization of some respiratory pathogens, including *K. pneumoniae*, by nonphagocytic cells such as the A549 epithelial cells, failed to promote subsequent invasive infection but rather represents an innate defense mechanism to contain the infection. The increased deposition of complement opsonic fragments at the surface of unencapsulated strains could explain the above observations [51]. It could also be considered that the internalization of *K. pneumoniae* triggers the inflammatory response and induces the recruitment of additional inflammatory cells to the airspace, thereby suppressing infection [50].

Loss of CPS is a common event observed within a bacterial population exposed to a capsule-targeting pressure, which is accompanied by the emergence of resistance. The consequences of mutation in the *wbaP* gene or its equivalent *wcaJ* in glucose-transferring *K. pneumoniae* strains, driven by phages equipped with virion-associated depolymerases, have been recently reported [31,52,53]. By analyzing the susceptibility to phages using different receptor types, the emergence of cross-resistance to depolymerase and phages targeting the same receptor was observed while simultaneously making resistant populations susceptible to infection by phage targeting an alternative one. This implies that during the design of therapeutic cocktails composed of depolymerases and phages, the priority is to select specificity rather than efficacy to efficiently kill pathogens. Importantly, although it has been reported earlier that the deletion of *wcaJ* in *K. pneumoniae* imparts resistance to quinolones [54], our study revealed that the mutation in *wbaP* did not affect drug susceptibility to quinolones, β-lactams, and aminoglycosides, as well as sulfonamide/trimethoprim.

In summary, it was confirmed that the application of phage-borne depolymerases targeting bacterial surface polysaccharides might be of great therapeutic interest as antivirulence agents: first, because of the direct degradation of bacterial protective shields, and second, by the selection of resistant mutants presenting a decreased potential for pathogenicity.

## 4. Materials and Methods

### 4.1. Bacterial Strains and Phages

*K. pneumoniae* strain 486 (Kp486) serotype K63, its mutant strain (Kp7De), and four *Klebsiella*-specific bacteriophages (Table S3) used in this study originate from the collections of the Department of Pathogen Biology and Immunology (Wroclaw, Poland). Unless indicated otherwise, bacteria were cultured in Trypticase Soy Broth or Agar (TSB or TSA, BioMerieux, Marcy-l’Etoile, France) at 37 °C. Phage titers and specificity were determined by a double-agar overlay plaque assay as previously described [55].

### 4.2. Recombinant Depolymerase Preparation

Recombinant capsule depolymerase KP36gp50 (NCBI acc. No. YP_009226010.1) encoded by phage KP36 was prepared as previously described (Majkowska-Skrobek et al. 2016). Briefly, PCR amplification product was cloned into the pEXP5-CT/TOPO^®^ expression vector (Invitrogen, Carlsbad, CA, USA) with a C-terminal His6 tag followed by transformation into *E. coli* BL21(DE3) strain. Recombinant protein expression was induced by 0.1 mM isopropyl-β-D-thiogalactopyranoside (IPTG). The His-tagged protein purification was performed with Bio-Scale Mini Profinity IMAC cartridges (Bio-Rad, Dreieich, Germany) on an FPLC-system (Bio-Rad, Hercules, CA, USA) followed by dialysis against phosphate-buffered saline (PBS) buffer (137 mM NaCl, 2.7 mM KCl, 10 mM Na_2_HPO_4_, 1.8 mM KH_2_PO_4_, pH 7.4) using Float-A-Lyzer (Serva, Heilderberg, Germany). The activity determination and visualization of enzyme specificity was performed on bacterial lawns using a standard spot assay [27].

### 4.3. Selection of Depolymerase-Resistant Mutant

The selection of depolymerase-resistant mutants was performed on biofilm-forming *K. pneumoniae* culture according to the method described by Harrison et al. [56] with modifications. The experiment was initiated by placing the polystyrene pegs surfaces protruding down from the lid (NUNC, Thermo Fisher Scientific, Roskilde, Denmark) in the wells of a 96-well microtiter plate (NUNC, Thermo Fisher Scientific, Denmark) containing 150 µL bacterial culture per well, which corresponds to approximately 10^6^ CFU/peg. After 24 h of incubation at 37 °C, the biofilm-containing pegs were rinsed and transferred to a standard 96-well plate containing 200 µL of KP36gp50 depolymerase solution (7 µg/mL) for another 24 h at 37 °C. Afterwards, biofilms were rinsed again and placed in a microtiter “recovery” plate that contained 200 µL of PBS in each well. Sessile cells were recovered from pegs by sonication at 42 kHz in a water bath sonicator (POLSONIC model Sonic3, Warsaw, Poland) for 15 min. Suspensions were subsequently transferred to Eppendorf tubes and vortexed vigorously for 30 s. Finally, bacteria were plated on ten-fold serial dilutions onto TSA plates, which was followed by overnight incubation at 37 °C. The discrete colonies per plate were picked to obtain resistant mutants. The sensitivity of bacterial mutants and the wild-type strain to KP36gp50 depolymerase and phage KP36 was verified by a spot assay on double-layer plates. The assay was carried out directly after the isolation of resistant bacterial clones and five successive passages on TSA to confirm the stability of the resistant phenotypes through multiple generations.

### 4.4. Genome Sequence Modification Analyses

The genomes of Kp486 and Kp7De have been made available via the NCBI BioProject database (PRJNA631924) [31]. The Kp486 wild-type genomic DNA (gDNA) was sequenced by the hybrid method (PacBio RSII (Pacific Biosciences, Inc., Macrogen, Menlo Park, CA, USA) + HiSeq (Illumina, Inc., San Diego, CA, USA) and Illumina HiSeq (Illumina, Inc.) was used for the mutant (Kp7De) genome. The single nucleotide polymorphisms (SNPs) and insertions/deletions (indels) were analyzed using Snippy v4.3.8 [57]. Furthermore, assembled genomes of strains Kp486 and Kp7De were typed for *Klebsiella* K and O loci by the Kaptive Web algorithm (https://github.com/katholt/Kaptive, Kaptive v0.7.3, 20 January 2021) [32].

### 4.5. Carbohydrate Surface Antigens Isolation

For surface polysaccharide extraction according to Bales’s protocol [58], the three-day bacterial cultures in TSB were used. Briefly, following the addition of 60 µL formaldehyde to every 10 mL of culture for 1 h and then 1 M NaOH at a ratio of 1:2.5 (*v*/*v*) for 3 h at RT, with agitation, the secreted polysaccharides were separated from bacterial cells by centrifugation (16,800× *g*, 1 h, 4 °C). The supernatant containing soluble polysaccharides was filtered through a 0.22 µm filter (Millipore, Darmstadt, Germany) and dialyzed against an excess of MilliQ water using a 12–14 kDa MWCO membrane (Serva, Heidelberg, Germany) for 24 h at 4 °C. Subsequently, trichloroacetic acid (TCA; 20% *w*/*v*) was added to the supernatant and centrifuged (16,800× *g*, 1 h, 4 °C) after 30 min of incubation on ice. The pellet was discarded, and then, polysaccharides remaining in the supernatants were precipitated from lipids by adding 1.5 volumes of 96% ethanol at −20 °C for 24 h. The precipitate was collected by centrifugation (16,800× *g*, 1 h, 4 °C) and resuspended in MilliQ water. Following the second dialysis against MilliQ water, the remaining retentate was lyophilized overnight and stored at 4 °C for further analyses. Crude polysaccharides were fractionated by gel chromatography on TSKgel G3000PW column (2.15 × 60 cm, Tosoh Bioscience LLC, Leuschnerpark, Germany) using water as eluent. The 1 mL fractions were collected, combined according to differential refractometric detector and UV λ = 190 nm measurements, and freeze-dried.

For LPS structural analyses of Kp486 and Kp7De, the bacterial cultures were routinely grown in 1 l of Lysogeny Broth proceeded by agar plate overnight culture to obtain 10 mL of inoculum. Strains were cultured for 24 h at 37 °C with agitation and then killed with 3% formalin (*v*/*v*) overnight. Samples were centrifuged, washed, suspended in water, and freeze-dried.

LPS was extracted from bacterial cells by the hot phenol/water method [59]. The water phase was dialyzed against deionized water for 3 days (ZuluTrans, 14 kDa MWCO; Roth, Germany) and freeze-dried. The crude LPS was resuspended in ultrapure water, homogenized by sonication, and purified by ultracentrifugation three times for 6 h at 105,000× *g*, 4 °C. The O-specific polysaccharide (O-PS) was isolated from LPS (2–5 mg) by treatment with 1.5% acetic acid (1 mL) at 100 °C for 45 min. The reaction mixture was centrifuged to separate lipid A sediment from soluble (LPS-derived poly- and oligosaccharides). Freeze-dried supernatant was fractionated by gel chromatography on TSKgel G2500PW column (7.5 × 60 cm, Tosoh Bioscience LLC, Leuschnerpark, Germany) using water as eluent. The 0.5 mL fractions were collected, combined according to differential refractometric detector and UV λ = 190 nm measurements, and freeze-dried.

*K. pneumoniae* reference LPS and O-specific polysaccharides O1 variant 1 and 2 isolated from strains PCM 063 and Kp24, O2a, O2afg, O2aeh, O3, O3a, O3b, O4, O5, O7, O8, and O12 were obtained from the collection of the Laboratory of Microbial Immunochemistry and Vaccines and Polish Collection of Microorganisms (Ludwik Hirszfeld Institute of Immunology and Experimental Therapy, PAS, Wroclaw, Poland).

### 4.6. Structural Analysis of Surface Polysaccharides

CPS and O-PS were analyzed by NMR spectroscopy using an Avance III 600 MHz (Bruker BioSpin, Rheinstetten, Germany) spectrometer equipped with a 5 mm QCI cryoprobe with *z*-gradient. Carbohydrate samples were analyzed in ^2^H_2_O using acetone as an internal reference (*δ*_H_/*δ*_C_ 2.225/ 31.05 ppm), processed, and analyzed as described previously [60]. The spectra were acquired and processed with the help of standard Bruker software (Bruker BioSpin GmbH, Rheinstetten, Germany). The 2D spectra were assigned using the NMRFAM-SPARKY program [61].

### 4.7. Bacterial Growth Curve Determination

The growth kinetics of the mutant and its parental strain was determined by monitoring culture optical density (OD at 600 nm) for 24 h at 37 °C (VarioSkan™ LUX, Thermo Scientific, Vantaa, Finland). Bacterial cultures with the starting density of 5 × 10^5^ CFU/mL were grown in a flat-bottomed 96-well microplate (Thermo Fisher Scientific, Roskilde,, Denmark), with a shaking cycle every 30 min when OD was measured. All assays were repeated at least three times.

### 4.8. Antibiotics Susceptibility Testing

The antibiotic susceptibility of bacterial strains was evaluated using the disk diffusion technique on Mueller–Hinton agar plates (BioMerieux, Marcy-l’Etoile, France) using: amoxicillin + clavulanic acid (AMC); piperacillin + tazobactam (TZP); cefuroxime (CXM); cefotaxime (CTX); ceftazidime (CAZ); aztreonam (ATM); meropenem (MEM); gentamycin (GM); ciprofloxacin (CIP); and trimethoprim + sulfamethoxazole (SXT), following the European Committee on Antimicrobial Susceptibility Testing (EUCAST) recommendations (https://www.eucast.org/ast_of_bacteria/). The minimum inhibitory concentrations (MICs) were interpreted according to EUCAST Enterobacterales calibration of zone diameter breakpoints to MIC values (Version 8.0, January 2020). The colistin (CS) MICs determination was performed by broth microdilution utilizing ComASP Colistin 0.25–16 μg/mL assay (Liofilchem, Roseto degli Abruzzi, Italy). The ability to produce extended-spectrum beta-lactamases (ESBLs) was analyzed according to guidelines (EUCAST breakpoint tables, version 8.1, 11 February 2018). The resistance profile was assigned for MICs: AMC > 8 mg/L; TZP > 16 mg/L; CXM > 8 mg/L; CTX > 2 mg/L; CAZ > 4 mg/L; ATM > 4 mg/L; MEM > 8 mg/L; CIP > 0.5 mg/L; GM > 8 mg/L; SXT > 4 mg/L; CS > 2 mg/L.

### 4.9. Serum Resistance Assay

The resistance of *K. pneumoniae* clones to serum bactericidal activity was determined in 50% pooled Normal Human Serum (NHS) purchased from the Regional Center of Transfusion Medicine and Blood Bank (Wroclaw, Poland) and stored at −80 °C. It was conducted according to the principles expressed in the Law on the public service of the blood of May 20, 2016 and in the Directive 2002/98/EC of the European Parliament and of the Council of 27 January 2003, establishing standards of quality and safety for the collection, testing, processing, storage, and distribution of human blood and blood components. Briefly, ≈2 × 10^6^ CFU of log-phase bacteria were suspended in PBS and mixed at a 1:1 *v*/*v* ratio with either NHS or heat-inactivated NHS (56 °C, 30 min). The mixture in final volume of 200 µL was incubated for 3 h at 37 °C, and at 0, 1, 2, and 3 h intervals; then, 20 µL aliquots were removed, diluted, and cultured on TSA for colony enumeration. Each test was performed at least in three independent experiments.

### 4.10. Phagocytosis Assays

Phagocytosis assays were performed as described previously [28]. Briefly, Human monocyte/macrophage cell line THP1 (ATCC, TIB-202) was maintained in RPMI-1640 medium (Lonza, Verviers, Belgium) supplemented with 10% heat-inactivated fetal bovine serum (HIFBS; GIBCO, Life Technologies, Grand Island, NY, USA), 1× glutaMAX (GIBCO, Life Technologies, Grand Island, NY, USA), and 1× antibiotic–antimycotic solution (GIBCO, Life Technologies, Grand Island, NY, USA) at 37 °C in a 5% CO_2_ atmosphere.

(i) Fluorescence labeling of *Klebsiella* strains

Washed bacteria (ca. 10^9^ CFU) were suspended in 200 µL of PBS. After 2 h incubation at 37 °C, bacteria were harvested by centrifugation at 5000× *g* for 15 min at 4 °C and resuspended in PBS. For fluorescent labeling, bacteria were killed by UV for 1 h (Osram Germicidal Puritec HNS 30W G13, Saint Petersburg, Russia) and subsequently incubated with 1 mg/mL of fluorescein isothiocyanate (FITC, Thermo Scientific, Rockford, IL, USA) at 0.05 M carbonate/bicarbonate buffer (pH 9.5) at 37 °C for 30 min with gentle mixing in the dark. To remove the excess dye, FITC-conjugated bacteria were washed twice with ice-cold carbonate/bicarbonate buffer, resuspended in PBS, and stored at −80 °C until further use.

(ii) Uptake of FITC-labeled bacteria

First, fifty µL of UV-killed FITC-labeled bacteria in Hank’s Balanced Salt Solution Ca^2+^Mg^2+^ buffer (HBSS-Ca^2+^Mg^2+^; Lonza, Verviers, Belgium) supplemented with 1% HIFBS were added to 50 µL of non-adherent THP-1 cells (10^7^ cells/mL) in the same buffer to get an MOI of 100:1. After 2 h incubation at 37 °C in a humidified 5% CO_2_ atmosphere, the cells were placed on ice to halt phagocytosis and then treated with 0.2 mg/mL of trypan blue solution to quench extracellular fluorescence. As a negative phagocytic control, each bacterial strain and phagocytic cell combination was also kept on ice to block the endocytic uptake of bacteria. For analysis by flow cytometry on FACS Calibur (Becton Dickinson, Franklin Lakes, NJ, USA), the samples were diluted 1:1 in ice-cold HBSS-Ca^2+^Mg^2+^ followed by being washed twice in ice-cold HBSS-Ca^2+^Mg^2+^. Each assay was performed at least three times in duplicate. The THP-1 effector cells were selected according to their forward and side scatter properties using THP-1 cells alone as a control. Data from 10,000 events (cells) per condition were collected and analyzed using Flowing Software (version 2.5.1).

### 4.11. Adhesion Assay

Human lung epithelial cells A549 (ATCC, CCL-185^TM^) were maintained in RPMI-1640 medium supplemented with 10% heat-inactivated fetal bovine serum (HIFBS; GIBCO, Life Technologies), 1× glutaMAX (GIBCO, Life Technologies, Grand Island, NY, USA), and 1× antibiotic–antimycotic solution (GIBCO, Life Technologies, Grand Island, NY, USA) at 37 °C in a 5% CO_2_ atmosphere. For the adhesion experiment, A549 cells were seeded at an initial density of 5 × 10^5^ cells per well at a final volume of 1 mL in 24-well flat-bottom microplates (NUNC, Thermo Fisher Scientific, Roskilde, Denmark) and incubated under 5% CO_2_ at 37 °C for 24 h (≈90–95% confluence). Subsequently, after washing three times with warm PBS/ HBSS (1:1, *v*/*v*), the log phase bacteria in 500 μL of RPMI-1640 without antibiotics were added to each well in duplicate to get an MOI of 10, and the plate was incubated under 5% CO_2_ at 37 °C for 2 h. After five times washing with 1 mL of PBS each time, the bacteria were released by the addition of 500 µL of 0.05% trypsin/0.08% saponin solution (*v*/*v*) for 10 min with shaking. Then, the lysates were transferred to 1.5 mL Eppendorf tubes and vortexed vigorously for 30 s. The bacteria were plated in 10-fold serial dilutions onto TSA plates in triplicate and incubated overnight at 37 °C. Adhesion was expressed as the number of CFU adherent to eukaryotic cells per well. All assays were repeated four times.

### 4.12. Gentamicin Protection Assay

To evaluate the ability of *K. pneumoniae* to invade into epithelial cells, the infected monolayers of the A549 line were incubated under 5% CO_2_ at 37 °C for 2 h to allow bacterial entry. After incubation, extracellular and adherent bacteria were killed by an addition of 500 µL of 200 μg/mL gentamicin (MP Biomedicals, Santa Ana, CA, USA) in RPMI-1640 and incubated for 2 h or 24 h at 37 °C under 5% CO_2_. The drug was subsequently removed by PBS washing, and epithelial cells were lysed and recovered as described before. The resulting lysate was serially diluted in PBS and quantified by viable counts on TSA plates after 24 h.

### 4.13. Statistical Analysis

Statistical analyses were performed using GraphPad Prism version 6.0 (GraphPad Software Inc., La Jolla, CA, USA). Student’s t-test (two-tailed) was used to compare the uptake of FITC-labeled bacteria. The remaining data were analyzed using the Mann–Whitney U test. p-values less than 0.05 were considered statistically significant.

## Figures and Tables

**Table 1:** LPS and CPS identification and serotyping in *K. pneumoniae* Kp486 parental strain and Kp7De mutant.

*K. pneumoniae* Strain	O-Serotyping (by Kaptive ^a^)	O-Serotyping (by NMR)	K-Serotyping (by NMR)	K-Serotyping (by Kaptive)
Kp486	O1 variant 2	O1 variant 2	K63	K63
Kp7De	O1 variant 2	O1 variant 2	devoid of CPS	K63

^a^ Kaptive Webtool [32].

**Table 2:** ^1^H and ^13^C NMR chemical shifts and sugar connectivities of CPS K63 isolated from *K. pneumoniae* Kp486 ^a^.

Residue	Atom Chemical Shift (ppm)						Connectivities to δ_H_ δ_C_		Inter-Residue Atoms/Residues
	H1/C1	H2/C2	H3/C3	H4/C4	H5/C5	H6,6′/C6 (CH_3_)			
A →3)-α-D-Gal*p*	5.25 95.8	4.09 68.5	4.04 78.1	3.92 72.3	4.20 67.9	3.74 61.9	4.14	75.0	H-3, C-3 of B
B →3)-α-D-Gal*p*A-(1→	5.31 101.5	4.00 67.9	4.14 75.0	4.55 67.9	4.37 72.8	176.2	4.06	78.3	H-3, C-3 of C
C →3)-α-L-Fuc*p*-(1→	5.21 101.5	3.96 68.4	4.06 78.3	3.90 72.7	4.18 67.8	1.18 16.1	4.04	78.1	H-3, C-3 of A

^a^ Spectra were recorded for ^2^H_2_O solution at 298 K. Acetone (δ_H_/δ_C_ 2.225/31.05 ppm) was used as an internal reference.

## Data Availability

Not applicable.

## Supplementary Materials

The following are available online at https://www.mdpi.com/article/10.3390/ijms222111562/s1.
