# Supplementary material for: The Mutation in wbaP cps Gene Cluster Selected by Phage-Borne Depolymerase Abolishes Capsule Production and Diminishes the Virulence of Klebsiella pneumoniae
Source: Int J Mol Sci. 2021 Oct 26;22(21):11562. doi: 10.3390/ijms222111562 (PMC8583740; doi:10.3390/ijms222111562)
Supplement: Supplementary file 1 [file ijms-22-11562-s001.zip › ijms-1429341-supplementary.pdf]

International Journal of
Molecular Sciences

MDPI

Supplementary data

Table S1. Genetic changes present in the *K. pneumoniae* 7De mutant. No mutations were detected in the strain's plasmids.

Position in the bacterial chromosome	Reference nucleotide	Alteration nucleotide	Type of mutation	Gene	Product
1729903	AT	C	frameshift variant, start lost	*wbaP*	undecaprenyl-phosphate galactose phosphotransferase WbaP
2437981	G	C	synonymous variant	*ydcP_2*	putative protease YdcP
2797478	A	G	-	intergenic region	possible promoter of putative L-galactonate transporter
3080156	G	A	synonymous variant	*sapA*	peptide transport periplasmic protein SapA
3095136	C	A	synonymous variant	hypothetical gene	putative kinase
5018734	G	A	-	intergenic region	-

Table S2. ^{1}H and ^{13}C NMR chemical shifts and inter-residue connectivities of the O-specific polysaccharides from *K. pneumoniae* Kp486 (O1v1).

Residue	Atom chemical shift (ppm)						Connectivities to		Inter-Residue Atoms/Residues
	H1/C1	H2/C2	H3/C3	H4/C4	H5/C5	H6,6′/C6 (CH_3)	δ_H	δ_C	
**A** →3)-β-D-Gal*f*-(1→	5.22<br>110.5	4.35<br>81.4	4.09<br>85.3	4.31<br>80.5	3.86<br>71.0	3.69<br>63.8	3.92	77.7	H-3, C-3 of **C**
**B** →3)-α-D-Gal*p*-(1→	5.19<br>95.8	4.05<br>68.1	4.16<br>80.0	4.29<br>70.0	4.24<br>71.3	3.76<br>61.9	4.15	80.0	H-3, C-3 of **E**
**C** →3,4)-α-D-Gal*p*-(1→	5.10<br>100.8	4.10<br>68.7	3.92<br>77.7	4.18<br>79.1	4.24<br>71.4	3.79<br>60.9	4.09	85.3	H-3, C-3 of **A**
**D** →D-Gal*p*-(1→	5.01<br>101.2	3.83<br>69.9	3.92<br>70.0	4.07<br>69.5	4.24<br>71.4	3.79<br>60.9	4.18	79.1	H-4, C-4 of **C**
**E** →3)-β-D-Gal*p*-(1→	4.69<br>105.2	3.75<br>70.5	3.81<br>77.7	4.19<br>65.6	3.68<br>75.6	3.76<br>61.9	4.16	80.0	H-3, C-3 of **B**

Spectra were recorded for $^{2}H_2O$ solution at 298 K. Acetone (δ_H/δ_C 2.225/31.05 ppm) was used as an internal reference.

Table S3. Characteristics of the four *Klebsiella*-specific phages used in this study.

	vB_KpnS_KP36 [phage KP36]	vB_KpnP_KP34 [phage KP34]	vB_KpnM_KP27 [phage KP27]	vB_KpnM_KP15 [phage KP15]
Genus	*Webervirus*	*Drulisvirus*	*Slopekvirus*	*Slopekvirus*
Host strain	Kp486	Kp486	KpATCC 700603<br>Kp767	KpATCC 700603<br>Kp767
Capsular-type specificity	K63	K63	KL53<br>KL10	KL53<br>KL10
GenBank accession number	NC_029099.1	NC_013649.2	NC_020080.1	NC_014036.1

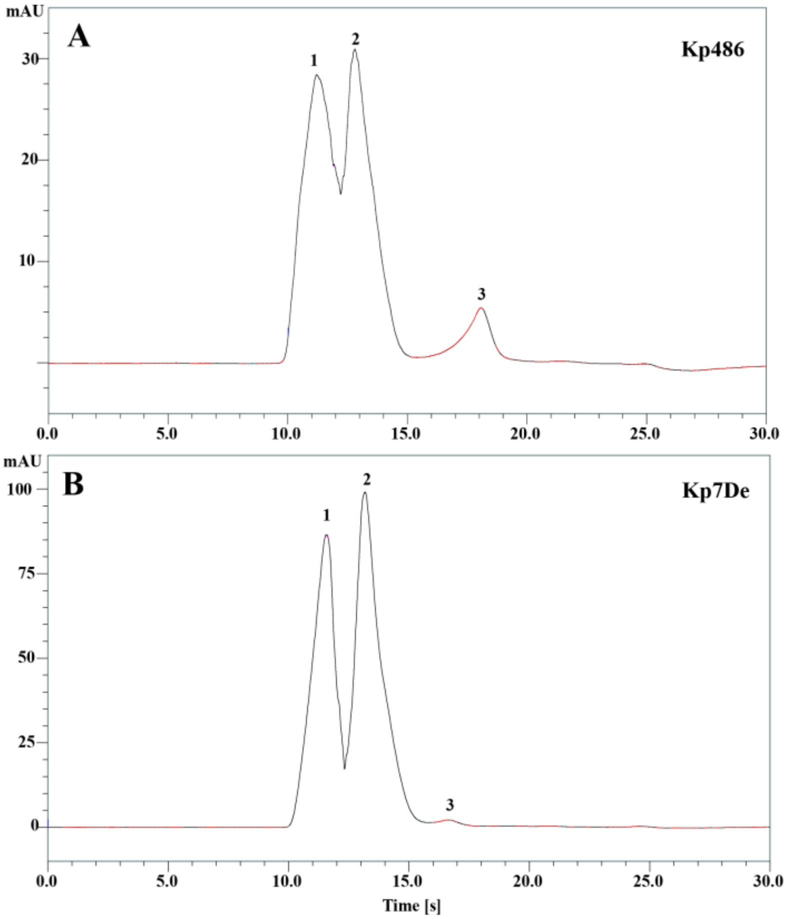

Figure S1. Comparison of elution profiles for poly- and oligosaccharides isolated by mild acid hydrolysis of Kp486 (A) and Kp7De (B) LPS. Numbers indicate fractions (1, 2, 3). Three fractions were eluted and checked by ^{1}H NMR spectroscopy. Fractions 1 did not contain sugar constituents, fractions 2 were identified as O-specific polysaccharide (O-PS), and fractions 3 as unsubstituted core oligosaccharides of LPS.
